# Alginate Nanoencapsulated Synbiotic Composite of Pomegranate Peel Phytogenics and Multi-Probiotic Species as a Potential Feed Additive: Physicochemical, Antioxidant, and Antimicrobial Activities

**DOI:** 10.3390/ani13152432

**Published:** 2023-07-27

**Authors:** Nesrein M. Hashem, Nourhan S. Hosny, Nagwa El-Desoky, Yosra A. Soltan, Ahmed A. Elolimy, Sobhy M. A. Sallam, El-Sayed M. Abu-Tor

**Affiliations:** 1 Animal and Fish Production Department, Faculty of Agriculture (El-Shatby), Alexandria University, Alexandria 21545, Egypt; enagwa278@gmail.com (N.E.-D.); yosra.soltan@alexu.edu.eg (Y.A.S.); soubhy.salam@alexu.edu.eg (S.M.A.S.); 2 Livestock Research Department, Arid Lands Cultivation Research Institute, City of Scientific Research and Technological Applications (SRTA-City), Alexandria 21934, Egypt; nourhansaad80@yahoo.com; 3 Animal Production Department, National Research Centre, Giza 12622, Egypt; elolimy@yahoo.com; 4 Food Science and Technology Department, Faculty of Agriculture (El-Shatby), Alexandria University, Alexandria 21545, Egypt; abutor_nesr@yahoo.com

**Keywords:** nanoencapsulation, synbiotic, antimicrobial activity, antibiotic alternative

## Abstract

**Simple Summary:**

Producing antibiotic-free animal products is one of the urgent global concerns, particularly the emergence of antimicrobial resistance, which has caused several health problems in humans and animals. Inventing natural antibiotic alternatives with multi-biological functions can present a good solution for sustainable animal production without additional load on human and animal health. The integration of biological and industrial technologies, such as microbiology, extraction of phytogenics, and nanotechnology, can aid in innovating new eco-friendly feed additives that can not only act as antibiotic alternatives but may also improve the general health of animals. Therefore, this study was designed to evaluate the physicochemical, antioxidant, and antimicrobial activities of a newly innovated alginate nanoencapsulated synbiotic composite of pomegranate peel phytogenics and multi-probiotic species as a potential feed additive.

**Abstract:**

A synbiotic composed of alginate nanoencapsulated prebiotic (pomegranate peel phytogenics) and multi-species probiotics (*Lactococcus lactis*, *Lactobacillus plantarum*, *Lactobacillus paracasei*, and *Saccharomyces cerevisiae*) has been developed as a potential eco-friendly alternative to antibiotics. The physicochemical properties of the encapsulated synbiotic were evaluated, and its gastric and storage tolerance, as well as its antioxidant and antimicrobial activity, were tested and compared to that of the non-encapsulated synbiotic (free synbiotic). The results showed that the prebiotic pomegranate peel ethanolic extract contained seven phenolic compounds, with cinnamic being the most abundant (13.26 µL/mL). Sodium alginate-CaCl_2_ nanocapsules were effective in encapsulating 84.06 ± 1.5% of the prebiotic’s phenolic compounds and 98.85 ± 0.57% of the probiotics. The particle size of the alginate-CaCl_2_ nanoencapsulated synbiotic was 544.5 nm, and the polydispersity index and zeta potential values were 0.593 and −12.3 mV, respectively. Thermogravimetric analysis showed that the alginate-CaCl_2_ nanoencapsulated synbiotic had high thermal stability at high temperatures, with only 2.31% of its weight being lost within the temperature range of 70–100 °C. The count of viable probiotics in the nanoencapsulated synbiotic was significantly higher than that in the free synbiotic after exposure to gastric acidity and storage for six months at room temperature. The percent inhibition values of the nanoencapsulated synbiotic and ascorbic acid (as a standard antioxidant) were comparable and significantly greater than those of the free synbiotic. The half-maximal inhibitory concentrations (IC50) of the nanoencapsulated synbiotic and ascorbic acid were significantly lower than those of the free synbiotic (3.96 ± 0.42 µg/mL and 4.08 ± 0.79 µg/mL for nanoencapsulated synbiotic and ascorbic acid, respectively, vs. 65.75 ± 2.14 µg/mL for free synbiotic). The nanoencapsulated synbiotic showed the highest significant antimicrobial activity against *Escherichia coli* (ATCC 8739). Both the nanoencapsulated and free synbiotics showed antimicrobial activity against *Staphylococcus aureus* (ATCC 6538), similar to that of gentamicin, although the nanoencapsulated synbiotic showed significantly higher inhibition activity compared to the free synbiotic. The nanoencapsulated synbiotic showed antimicrobial activity comparable to gentamicin against *Pseudomonas aeruginosa* (ATCC 90274), whereas the free synbiotic showed the least antimicrobial activity (*p* < 0.05). Both synbiotics showed significantly higher antimicrobial activity against Salmonella typhi (ATCC 6539) than gentamicin. Both synbiotics showed antifungal activity against *Aspergillus niger* and *Aspergillus flavus*, with a stronger effect observed for the nanoencapsulated synbiotic. However, the activity of both synbiotics was significantly lower than that of fluconazole (an antifungal drug).

## 1. Introduction

The world population is growing rapidly. According to the United Nations Department of Economic and Social Affairs Population Division, it is projected to be over 9.8 billion people by 2050 (UN/DESA, 2017 available at: https://population.un.org/wpp/, (accessed on 8 May 2023)). This growing population adds a heavy burden on the agricultural sector and food production systems worldwide, livestock farming being one of those agricultural sectors. To meet the continuous ascending need for animal protein foods, antibiotics are intensively used in the livestock production chain as antimicrobial growth promoters, but the excessive and improper use of antibiotics derives from the emergence of antibiotic resistance phenomenon [1,2]. This phenomenon has turned out into a public concern that threatens both human and animal health. Thus, minimizing the use of antibiotics during the production cycle of food-producing animals becomes an imperative need for maintaining human and animal health. 

Synbiotics are nutritional supplements that comprise a mixture of prebiotic and probiotic ingredients. Biologically, synbiotics can confer a wide array of health benefits to the host. They mainly maintain intestine eubiosis; moreover, they possess antimicrobial, anti-inflammatory, antioxidant, and immunomodulatory activities. These benefits nominate synbiotics as an effective intervention to support animal health and give the opportunity to minimize the use of synthetic drugs/antibiotics [2,3]. The biological activity of synbiotics depends on several factors, such as the types of probiotics, probiotic tolerance to gastrointestinal conditions and its colonization capacity, prebiotics source, manufacturing procedure, and storage conditions. Commonly, microbial species, including *Lactobacillus* spp., *Streptococcus* spp., *Bacillus* spp., and *Saccharomyces* spp., are used as potential probiotics [4]. They can possess antimicrobial activity against enteric pathogens, produce several nutrients and micro-nutrients, and produce bacteriocins and other biologically active postbiotics such as short-chain fatty acids [5,6]. As each probiotic strain has its specific features and metabolites, several reports have highlighted the benefits of the use of multi-strain probiotics in one formula [7,8]. This appears to be effective against a wide range of pathogens and stimulates different biological processes in the host’s body [4,9]. The other composite of synbiotic ingredients is prebiotics. For the long term, insoluble carbohydrates have been considered the main source of prebiotics; however, recently, prebiotics have included a wide range of phytochemicals, including a group of plant-based chemicals (soluble fibers, polyphenols, and polyunsaturated fatty acids) [2]. These new sources of prebiotics have been proposed to boost probiotic growth and provide additional health benefits to the host [10]. For example, cocoa-derived flavanols have been found to stimulate lactic acid bacteria growth [11]. In this respect, pomegranate peel is one of the biomass agro-industrial byproducts which can be converted from waste to valuable products. Pomegranate peel is the richest part of the plant, with considerable concentrations of phenolic compounds, including phenolic acids, flavonoids, lignans, stilbenes, and hydrolyzable tannins, and, therefore, it has the most antioxidant and antimicrobial activity [12]. 

As mentioned above, the effectiveness of synbiotics does not only depend on their probiotic and prebiotic ingredients but also on the ability of these components to tolerate the harsh conditions of the gastrointestinal tract and the manufacturing process as well. For example, lactic acid bacteria (*Lactobacillus*, *Streptococcus*, *Enterococcus*, *Lactococcus*, and *Leuconostoc*); however, while they are common probiotic candidates, their survivability is challenged by stomach acidity (pH 2 unit) and hydrolytic enzymes in the stomach and small intestine, decreasing the colonization and competitive capacity of probiotic strains in the digestive system of the host [2]. Indeed, unconventional prebiotic sources such as polyphenols and fatty acids may possess instability in the gastrointestinal tract, weak absorption and cellular uptake, and instability during handling and storage as well. Hence, to improve the biological and industrial activity of synbiotics, delivery systems based on the use of polymers as coating materials are designed for encapsulation, protection, and controlling the release of probiotics and prebiotics. The encapsulation of synbiotic composites (probiotics and prebiotics) using biopolymers in a nanoform is a promising technology to prolong the viability and colonization capacity of probiotics and the absorption of prebiotics through the gastrointestinal tract, which may help improve the intestinal eubiosis [13]. Among polymers, alginate is a biodegradable and biocompatible polysaccharide copolymer that is available at a low cost. Thus, alginate-based nanocarriers seem to have several advantages, making them successful drug delivery systems among different biological systems [14]. 

This study aimed to assess the efficacy of a novel synbiotic formulation, which combines a prebiotic source from pomegranate peel ethanolic extract with a multi-strain probiotic mixture of lactic acid bacteria and yeast using nanoencapsulation technology with alginate as a coating material. The physicochemical properties of the synbiotic were examined, along with its antioxidant and antimicrobial activities, to determine its potential as a nutraceutical supplement or feed additive.

## 2. Materials and Methods

This study was carried out at the nanobiotechnology and microbiology laboratory (Nanoencapsulation and Animal Physiology Unit), Faculty of Agriculture, Animal and Fish Production Department, Alexandria University, Egypt. This study is part of a project entitled “Designing an industrial prototype for innovating microbial-based feed additives using nanoencapsulation technology for improving performance and immunity of farm animals”, funded by the Academy of Scientific Research and Technology (ASRT), Science and Technology Center (STC), Egypt. The project protocols and procedures were checked and approved by the Institutional Animal Care and Use Committee of Alexandria University, ALEXU-IACUC, No: AU-08-23-02-26-4-129. 

### 2.1. Fabrication and Characterization of Synbiotics

#### 2.1.1. Prebiotic Preparation and Identification of Phenolic Compounds

Dried pomegranate peels were milled through a 1 mm screen. Pomegranate peels (15 g/100 mL) were extracted using a hydroethanolic solution (70%, *v*/*v*) at 45 °C for 72 h. The extracts were filtered with Whatman No. 1 filter paper (Camlab, Cambridge, UK). The collected filtrates were evaporated to complete dryness at 45 °C. The residues were then stored at −20 °C, pending use. The collected filtrate was evaporated to complete dryness at 45 °C. The residues were then stored at −20 °C, pending use [15]. For phenolic compound analysis, all analytical chemicals were of gradient grade for the HPLC analysis. All chemicals and standards were purchased from Sigma-Aldrich^®^ (Merck KGaA, Darmstadt, Germany). The polyphenol profile of the plant extract was assessed using high-performance liquid chromatography (HPLC) (Agilent 1100) fitted with two LC pumps and a UV/Vis detector. C18 column (125 mm × 4.60 mm, 5 µm particle size; Agilent, Santa Clara, CA, USA). Phenolic compounds were separated by employing a mobile phase of two solvents, 0.1% methanol:phosphoric acid (50:50 *v*/*v*, isocratic mode). The flow rate was adjusted at 1.0 mL/min; the detector was set at 280 nm with the mobile phase. Chromatograms were obtained and analyzed using the Agilent ChemStation [16]. 

#### 2.1.2. Probiotic Strains 

The probiotic strains *Lactococcus lactis* ATCC 11454, *Lactobacillus plantarum*, ATCC 14917, *Lactobacillus paracasei* ATCC 334, and *Saccharomyces cerevisiae* (strain ATCC 204508/S288c) (MIRCEN, Microbiological Resource Center, Faculty of Agriculture, Ain Shams University) were selected as probiotic strains. According to the MIRCEN-provided information, the bacterial strains were isolated from Anchu mash, Formosa, pickled cabbage, and dairy products. *Saccharomyces cerevisiae* was purchased in a lyophilized form and was ready for use. For mass production of lactic acid bacteria strains, De Man, Rogosa, and Sharpe (MRS, Merck KGaA, Darmstadt, Germany), agar and broth media were autoclaved at 120 °C and at 1–1.5 atm for 15 min. Each strain of lactic acid bacteria was grown in De MRS agar plates at 37 °C for 48 h. Then, the resultant colonies of each strain were inoculated in MRS broth under anaerobic conditions at 37 °C for 48 h. Wet biomass was harvested by centrifugation at 5000 rpm for 15 min at 25 °C. The bacterial biomass was washed three times with saline and re-suspended in MRS broth plus 15% glycerol (*v*/*v*) and kept at −80 °C. 

#### 2.1.3. Fabrication of Alginate-CaCl_2_-Based Nanosynbiotic and Free Synbiotics

Both sodium alginate (Oxford La Fine Chem Lip, Maharashtra, India) and calcium chloride (CaCl_2;_ Cheajet, Alexandria, Egypt) were used to fabricate the coating wall of the synbiotic, adopting the ionic-gelation method as described by Hashem et al. (2021) [2]. In brief, under continuous magnetic stirring, the pomegranate peel ethanolic extract (1.5 g), 10^12^ CFU/mL of each probiotic strain, 0.5% Tween 80 were first mixed with 100 mL sodium alginate solution (3%, *w*/*v*). Then, the mixture was added dropwise using a syringe pump into a 50 mL CaCl_2_ solution (2.22 mol/L). The synthesized nanoparticles were centrifuged at 8000 rpm for 20 min, and the resultant nanoparticles were collected and stored at −80 °C. Free, non-encapsulated synbiotic was fabricated using the previously mentioned prebiotic and probiotic strains but without encapsulation with alginate-CaCl_2_ as a coating material.

### 2.2. Synbiotic Characteristics

#### 2.2.1. Encapsulation Efficiency

The encapsulation efficiency (EE, %) of sodium alginate-CaCl_2_ nanoparticles for the synbiotic composite was estimated. For probiotics, EE was estimated as a percentage of the number of microbial cells in the supernatant to the number of microbial cells used during encapsulation. For prebiotic, the phenolic content of the raw extract (before encapsulation, BE) and of resultant supernatant following collection of the nanocomposite particles (C supernatant), using the following equation: EE (%) = BE − C supernatant/BE × 100, was used. The concentrations of individual detected phenolic compounds in the raw plant extract (BE) and the supernatant were performed by HPLC-(Agilent 1100).

#### 2.2.2. Morphology of Nanoencapsulated Synbiotics

Distribution of fabricated synbiotic nanoparticles was observed under Transmission Electron Microscope (JEM-2100Plus, JEOL Ltd., Akishima, Tokyo, Japan). One diluted drop of fabricated synbiotic nanoparticles suspension was deposited on a film-coated copper grid, and it was stained with one drop of 2% (*w*/*v*) aqueous solution of phosphotungstic acid. The excess solution was drained off with filter paper, and then, the grid was allowed to dry for contrast enhancement. The sample was then examined by Transmission Electron Microscopy.

#### 2.2.3. Size Distribution and Surface Charge

The physicochemical characteristics, including size, polydispersity (PdI), and zeta potential of nanoencapsulated synbiotic, were measured using a Scientific Nanoparticle Analyzer (Zetasizer Nano ZS, Malvern Instruments Ltd., Worcestershire, UK) at 25 °C.

#### 2.2.4. Thermogravimetric Analysis (TGA Analysis)

Thermal analysis of the nanoencapsulated synbiotic was carried out by thermogravimetric analysis (TGA) using Mettler Toledo TGA/SDTA 851e equipment. Samples (around 5 mg) placed in 70 μL platinum pans were heated in nitrogen with a 40 mL/min flow rate. The heating rate was 10 °C/min with a temperature range from 25 to 700 °C. 

#### 2.2.5. Fourier Transform Infrared Analysis

Fourier transform infrared (FTIR; Shimadzu-8400S, Osaka, Japan) spectrum analysis was carried out to identify functional groups of coating materials before and after gelation and after encapsulating prebiotics and probiotics.

### 2.3. Gastric Tolerance and Storage Survivability of Probiotics

The survivability of probiotics after incubation in simulated gastric juice (SGJ) was evaluated for free and nanoencapsulated synbiotics. Porcine pepsin (Sigma-Aldrich CHEMIE Gmbh, Steinheim, Germany) prepared with SGJ electrolyte solution and adjusted to 3.0 with HCl was used as simulated gastric fluid. A weight of 250 ug of the synbiotics was mixed with 3 mL of SGJ and kept in a shaking incubator at 100 rpm at 37 °C for 2 h. The viable cell count was assessed using the surface plate count method. The survivability of probiotics after storage of the free and nanoencapsulated synbiotics for six months at room temperature (25 °C) was evaluated using the surface plate count method [17]. The experiment was repeated three times for each synbiotic product.

### 2.4. Antioxidant Activity of the Synbiotics (DPPH Scavenging Activity)

Free radical scavenging activity of different extracts of the products was measured with 1, 1- diphenyl-2-picryl-hydrazyl (DPPH). In brief, DPPH ethanolic solution (0.1 mM) was prepared. This solution (1 mL) was added to 3 mL of different concentrations of each product (3.9, 7.8, 15.62, 31.25, 62.5, 125, 250, 500, 1000 μg/mL). The mixture was shaken vigorously and allowed to stand at room temp for 30 min. Then, absorbance was measured at 517 nm by using a spectrophotometer (UV-VIS Milton Roy, SpectraLab Scientific Inc., Markham, ON, Canada) [18]. The DPPH activity of the products was compared to ascorbic acid (L-ascorbic acid, 99% purity, Merck KGaA, Darmstadt, Germany) as a reference antioxidant. The analyses were performed in triplicate. The IC-50 values (the concentration of sample required to inhibit 50% of the DPPH free radical) of the samples were calculated using the log dose inhibition curve. The DPPH scavenging activity was calculated using the following equation: DPPH scavenging activity (%) = A1 − A2/A2 × 100, where A1 is the absorbance of the control reaction, and A2 is the absorbance in the presence of test or standard sample.

### 2.5. Antimicrobial Activity of the Synbiotics

**Figure 1:**
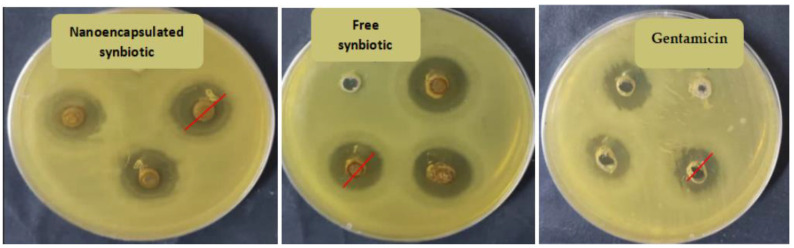
Representative plates for the antimicrobial activity of nanoencapsulated and free synbiotics and antibiotic gentamicin against *Staphylococcus aureus* (ATCC 6538). Red lines indicate the diameters of inhibition zones.

The antimicrobial activities of the samples were tested on Mueller–Hinton agar plates by the agar diffusion technique against four pathogenic bacterial strains and two fungal strains. Gentamicin and fluconazole (10 mg/mL) were used as the standard antibacterial and antifungal agents, respectively. A volume of 100 µL of the antibacterial and antifungal antibiotics and the products was added to a 6.0 mm-diameter well with Mueller–Hinton agar and Malt Extract agar plates seeded with 1.8 × 10^8^ CFU/mL of the tested pathogens. Following the 24-h incubation at 37 °C, plates were examined for the presence of inhibition zones. The inhibition zones surrounding the wells were measured (mm) considering only halos of >6 mm; inhibition zones obtained are the mean of three replicates for each experiment (Figure 1).

### 2.6. Statistical Analyses

The Statistical Package for the Social Sciences (SPSS, Version 26) was used for analyzing the results of the present study. The Generalized Linear Model (GLM) method uses the following model: yij = µ + Ti + eij, in which yij = the observed value of the dependent variable; µ = the overall mean; Ti = the fixed effect of the ith treatment, and eij = the residual error. Comparisons between treatment means were performed using Duncan’s multiple-range test. All results were expressed as the mean ± SEM. Significance was set at *p* < 0.05.

## 3. Results

### 3.1. Phenolic Compound of Prebiotic

**Figure 2:**
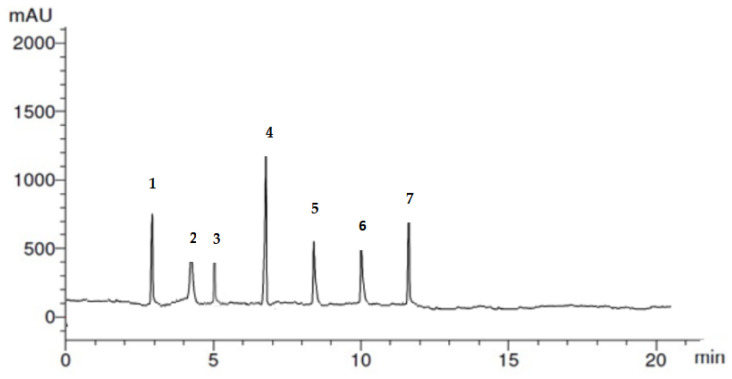
Absorbance (mAu) and retention time (min) of detected phenolic compounds of (prebiotic pomegranate peel phytogenics extract). The identified compounds ranked according to retention time are as follows: 1: chlorogenic; 2: catechol; 3: syringenic; 4: cinnamic; 5: caffeic; 6: gallic; and 7: salicylic acid.

The phenolic profile of the pomegranate peels ethanolic extract detected by HPLC is shown in Figure 2 and Table 1. A total of seven phenolic compounds were identified; the most abundant phenolic compound was cinnamic (13.26 µL/mL), followed by salicylic acid (5.36 µL/mL), chlorogenic (5.33 µL/mL), syringenic (4.68 µL/mL), and, lastly, catechol (3.1 µL/mL), caffeic (2.45 µL/mL), and gallic (2.19 µL/mL). 

### 3.2. The Physicochemical Characteristics of Nanoencapsulated Synbiotic 

**Figure 3:**
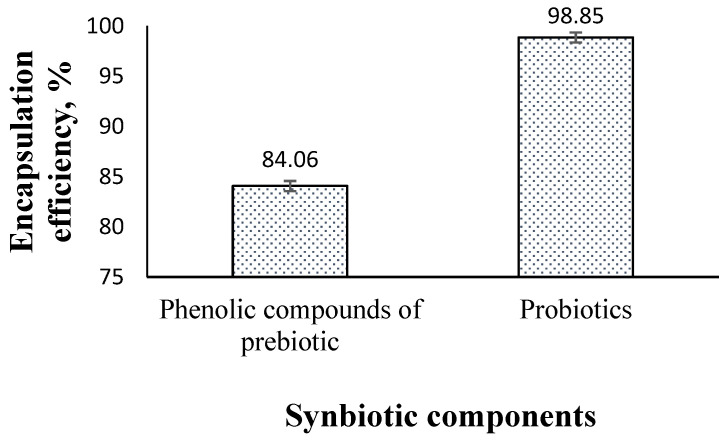
Encapsulation efficiency of alginate-CaCl_2_ for synbiotic components.

Results shown in Figure 3 revealed that sodium alginate-CaCl_2_ nanocapsules were effective in encapsulating 84.06% (±1.5) of phenolic compounds of the pomegranate ethanolic extract (prebiotic) and 98.85% (±0.57) of microbial cells (probiotics).

**Figure 4:**
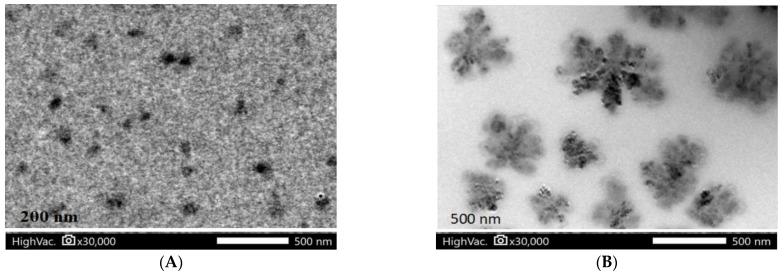
Morphology of alginate-CaCl_2_ nanocapsules (**A**) and synbiotic-alginate-CaCl_2_ nanocapsules (**B**) under transmission electron microscope.

Figure 4 shows the morphology of the alginate-CaCl_2_ and alginate-CaCl_2_ nanoencapsulated synbiotic using Transmission Electron Microscope. The images show that the alginate-CaCl_2_ particles showed a sphere-like shape with a homogenous, uniform, bead-free texture and an average particle size of 200 nm. Afterward, the alginate-CaCl_2_ nanoencapsulated synbiotic showed the same structure but with clusters of probiotic cells and a mean size of 500 nm.

**Figure 5:**
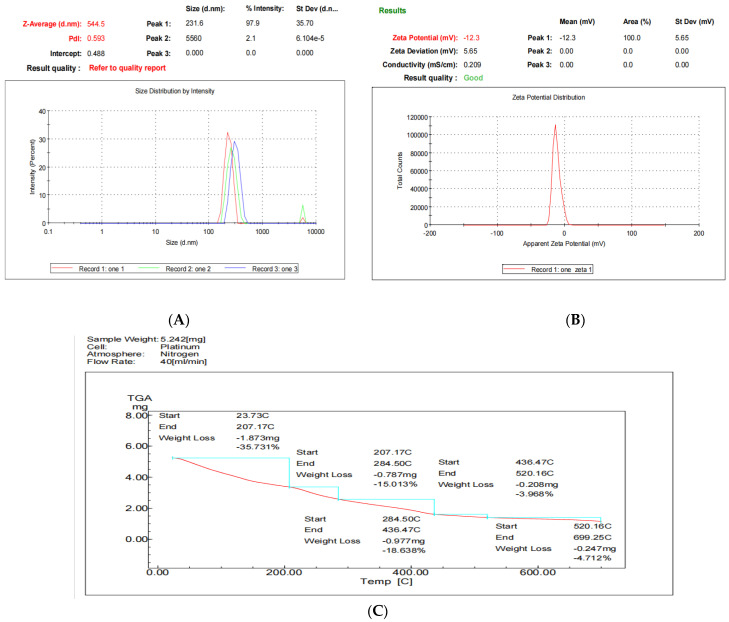
Size distribution and PdI (**A**), zeta potential (**B**), and thermogravimetric curve (**C**) of the nanoencapsulated alginate-CaCl_2_ synbiotic. Red line refers to the weight loss of the nanoencapsulated alginate-CaCl_2_ synbiotic at a temperature range between 25 to 700 °C. Blue lines refer to the weight loss of the nanoencapsulated alginate-CaCl_2_ synbiotic during specific temperature ranges.

The particle size analysis, using the nanosizer, for alginate-CaCl_2_ nanoencapsulated synbiotic showed that the mean size of alginate-CaCl_2_ nanoencapsulated synbiotic particles was 544.5 nm, and the PdI value was 0.593 (Figure 5A), matching the results of TEM images. The zeta potential value of alginate-CaCl_2_ nanoencapsulated synbiotic was −12.3 mV (Figure 5B). The thermogravimetric analysis showed high thermal stability of the alginate-CaCl_2_ nanoencapsulated synbiotic to high temperature. 

The product did not lose more than 2.31% of its weight at a temperature range between 70 and 100 °C, the pelleting temperature of the animal diets. The maximum percentage of weight loss was around 35.73% at 207.17 °C (Figure 5C). 

**Figure 6:**
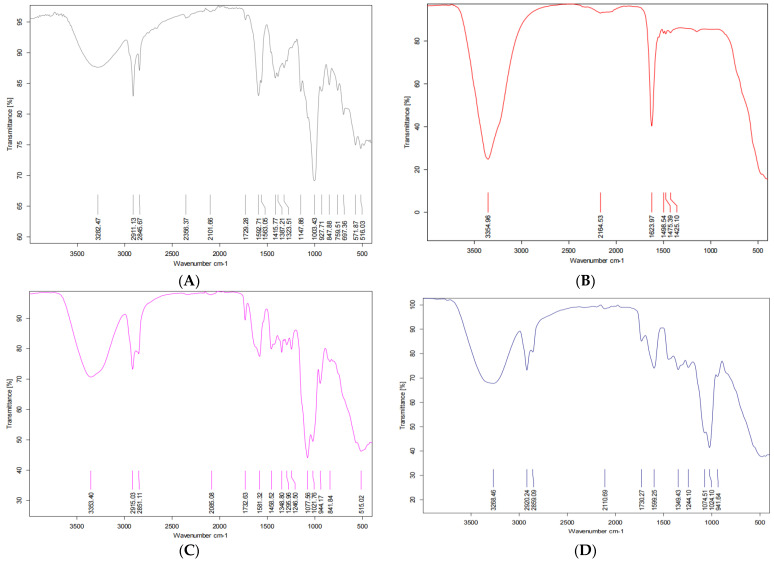
Fourier transform infrared spectroscopy (FTIR) analysis for the alginate (**A**), CaCl_2_ (**B**), alginate-CaCl_2_ complex (**C**), and nanoencapsulated alginate-CaCl_2_-synbiotic (**D**).

Figure 6A–D shows the FTIR analysis of alginate, CaCl_2_, alginate-CaCl_2_ complex, and alginate-CaCl_2_ nanoencapsulated synbiotic. The main functional groups of alginate are OH and C=O; these functional groups exert IR absorption at 3200–3550 cm^−1^ and 1680–1750 cm^−1^, respectively. The spectra of alginate-CaCl_2_ showed a change in the wavenumber of the C=O group of alginate after biding with CaCl_2._ Differences between alginate and alginate-CaCl_2_ complex in wavenumber and transmission intensity were observed for the OH and C-C functional groups. Alginate-CaCl_2_ complex nanoparticles showed different patterns of transmission intensity compared to those containing prebiotics and probiotics in OH (3230–3550 cm^−1^), C-C (750–1100 cm^−1^), and C-H (2850–3300 cm^−1^) bonds.

### 3.3. Gastric Tolerance and Storage Survivability of Probiotics

**Figure 7:**
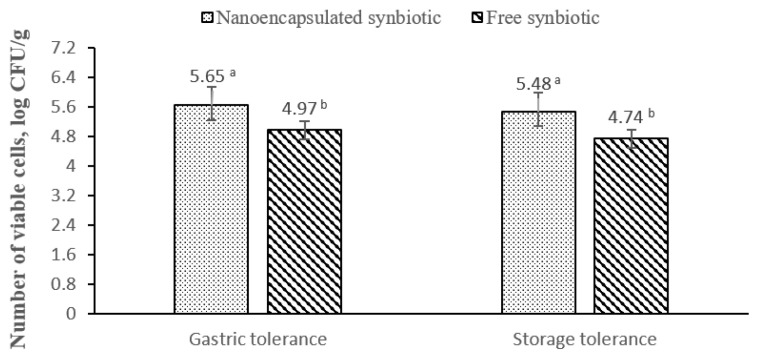
Gastric and storage tolerance of probiotics of nanoencapsulated and free synbiotics expressed as number of viable probiotic cells (Log CFU/g). Variable means with a and b superscript letters are significantly different (*p* < 0.05).

Figure 7 shows the count of viable probiotics of free and nanoencapsulated synbiotics following exposure to SGJ and storage for six months at room temperature (25 °C). The count of viable probiotics of nanoencapsulated synbiotics was significantly higher than those of free synbiotics after exposure to the acidity of SGJ and storage for six months at room temperature.

### 3.4. Antioxidant Activity of the Synbiotics

The antioxidant capacity (scavenging activity) of the nanoencapsulated and free synbiotics, as determined by the DPPH colorimetric test, is shown in Table 2. The percent inhibition values of the nanoencapsulated synbiotic and ascorbic acid (as standard antioxidants) were comparable and significantly greater than those of the free synbiotic. The half-maximal inhibitory concentration (IC50) of the nanoencapsulated and ascorbic acid was significantly lower than that of free synbiotic (3.96 ± 0.42 µg/mL and 4.08 ± 0.79 µg/mL for nanoencapsulated synbiotic and ascorbic acid, respectively, vs. 65.75 ± 2.14 µg/mL for free synbiotic).

### 3.5. Antimicrobial Activity of the Synbiotics

Results shown in Table 3 revealed that both nanoencapsulated and free synbiotics have antimicrobial activities against the tested microbial pathogenic strains and fungal strains. Nanoencapsulated synbiotics showed the highest significant antimicrobial activity against *Escherichia coli* (ATCC 8739). Both nanoencapsulated and free synbiotics showed antimicrobial activity against *Staphylococcus aureus* (ATCC 6538), similar to gentamicin (antibacterial drug); however, nanoencapsulated synbiotics showed higher significant inhibition activity compared to free synbiotics. The nanoencapsulated synbiotic showed antimicrobial activity comparable to gentamicin against *Pseudomonas aeruginosa* (ATCC 90274), whereas the free synbiotic showed the least antimicrobial activity (*p* < 0.05). Both synbiotics showed significantly higher antimicrobial activity against *Salmonella typhi* (ATCC 6539) than gentamicin. Both synbiotics showed antifungal activity against *Aspergillus niger* and *Aspergillus flavus*, with a stronger effect for nanoencapsulated synbiotics. However, the activity of both synbiotics was significantly lower than fluconazole (antifungal drug). 

## 4. Discussion

In our study, we aimed to create a synbiotic product that combines multiple microbial species, a novel prebiotic source (pomegranate peel extract), and nanoencapsulation technology to achieve unique biological and industrial properties. Such products are currently receiving increased attention as potential solutions for enhancing farm animal health and productivity while reducing reliance on synthetic growth promoters and antibiotics [2,3].

The encapsulation of synbiotic composites using alginate-CaCl_2_ nanocapsules as a coating material showed high encapsulation efficiency for both the prebiotics (approximately 85%) and probiotics (approximately 99%). These findings demonstrate the effectiveness of alginate-CaCl_2_ nanocapsules for encapsulating/coating synbiotic composites. The observed encapsulation efficiency of alginate-CaCl_2_ nanocapsules in this study is higher than that reported in other studies, which may be attributed to the phenolic compounds present in the pomegranate peel extract (prebiotic) [2,3]. Wu and Zhang [3] also found that the presence of a high ratio of ferulic acid, a phenolic acid found in prebiotic arabinoxylan, could improve the gelation properties of alginate-CaCl_2_ when used to encapsulate probiotic *Lactobacillus plantarum*.

In this study, the encapsulation of synbiotic composites using alginate-CaCl_2_ nanocapsules resulted in an average size increase from 200 nm (free nanocapsules) to 500 nm (synbiotic nanocapsules). Similar size increases have been observed in previous studies utilizing alginate-based nanoparticles for probiotic encapsulation. For instance, Duman and Karadag [19] used electrospun alginate-based nanofibers to encapsulate *Lactobacillus fermentum* and observed an increase in size from 192.20 nm (alginate nanofibers without probiotics) to 500–900 nm (alginate nanofibers with *Lactobacillus fermentum*). Similarly, Atraki and Azizkhani [20] obtained alginate-based nanofibers with an average diameter of 295 nm using an electrospun technique, and the inclusion of multiple species of probiotics (*Lactobacillus acidophilus* (LA5), *Lactobacillus rhamnosus* 23,527 LGG, *Bifidobacterium bifidum*, and *Bifidobacterium animalis*) increased the size to 797 nm. It is worth noting that the size of the synbiotic product obtained in this study is smaller than those observed in previous studies that used the electrospinning technique. These findings suggest that the ionic gelation procedure used in this study is an efficient and cost-effective method for producing nanoparticles that meet both biological and industrial requirements for the nanoencapsulation of active components such as probiotics and prebiotics.

This study revealed that the alginate-CaCl_2_-synbiotic nanocapsules had a negative surface charge, as indicated by a zeta potential of −12.3 mV. This negative charge can primarily be attributed to the anionic biopolymer alginate, which contains multiple carboxylic acid groups. The detachment of protons from the acid moieties of sodium alginate and the presence of free carboxylic acid groups on the surface of alginate molecules [20] may contribute to the negative charge of the alginate-CaCl_2_-synbiotic nanocapsules. Additionally, the cell membranes of probiotics also possess a negative charge, which could account for the overall negative charge of the nanocapsule [21].

The physicochemical properties of the alginate-CaCl_2_ synbiotic nanocapsules, as determined in this study, are crucial for conferring biological activities that are effective in traversing the hostile gastrointestinal tract. The mucosa layer lining of the gastrointestinal tract is composed primarily of mucin protein, a highly glycosylated protein essential for mucosal barrier function and the absorption capacity of various particles through the digestive system [22]. Biopolymers with mucoadhesive properties are capable of providing prolonged contact with the intestinal mucosa, thereby increasing the absorption of loaded compounds. Alginate, among other biopolymers, possesses significant mucoadhesive capacity and can interact chemically and/or physically with mucin and other mucosal components [23]. The mucoadhesive capacity of biopolymer particles can be controlled by their physicochemical properties, and biopolymers with nanosize and high surface area-to-volume ratios have substantially higher mucoadhesive capacity than their original forms [13]. Additionally, the surface charge of a biopolymer plays a pivotal role in its mucoadhesive capacity, with negatively charged biopolymers demonstrating high mucoadhesive capacity due to electrostatic repulsions created by the negative electrical charge of mucous and particle surfaces. Anionic biopolymer nanoparticles have also been found to induce tight junction relaxation, increasing the intestinal permeability [24]. Overall, the low-magnitude negative charge and moderate hydrophilicity of the biopolymer nanoparticles produced in this study contribute to their ease in passing through the small intestinal mucosa layer [25]. These physicochemical properties are essential for conferring the synbiotic formula with suitable properties for exerting effective biological activities through the gastrointestinal tract.

Alginate is a linear water-soluble polysaccharide composed of irregular blocks of β-D-mannuronic acid (M) and 1–4 linked α-L-guluronic residues (G). The main functional groups of alginate are the hydroxyl (-OH) and carbonyl (C=O) groups of the carboxyl group of β-D-mannuronic acid and α-L-guluronic residues, respectively, which exhibit IR absorption at 3200–3550 cm^−1^ and 1680–1750 cm^−1^. The spectra of alginate-CaCl_2_ complexes demonstrated a shift in the wavenumber of the C=O group of alginate after binding with CaCl_2_. Additionally, differences in the wavenumber and transmission intensity of the -OH and C-C functional groups were observed between alginate and alginate-CaCl_2_ complexes, which could be attributed to the gelation process triggered by the interaction of divalent cations with blocks of guluronic acid at different polysaccharide chains. The Ca^2+^ cation interacts with two carboxyl groups from different polymer chains, resulting in the formation of the “egg-box” structure [26]. Free alginate-CaCl_2_ nanocapsules exhibited different patterns of transmission intensity compared to those of encapsulated synbiotic composites (probiotics and prebiotics) for -OH (3230–3550 cm^−1^), C-C (750–1100 cm^−1^), and C-H (2850–3300 cm^−1^) functional groups, indicating the formation of new bonds between the alginate-CaCl_2_ complex nanoparticles and the functional groups of prebiotics.

Furthermore, alginate-based encapsulation systems can protect the encapsulated compounds from enzymatic degradation and pH changes in the gastrointestinal tract. Alginate is considered to be a biocompatible and biodegradable polymer, making it a safe and sustainable material for encapsulation purposes. The alginate-CaCl_2_ synbiotic nanocapsules in this study also exhibited a controlled release pattern, which is advantageous for the delivery of bioactive compounds to target sites in the body. The controlled release of the synbiotic compounds can help enhance their efficacy and reduce any potential adverse effects that may arise from rapid release or high concentrations of the compounds in the body. Overall, the physicochemical properties of the alginate-CaCl_2_ synbiotic nanocapsules make them a promising candidate for use as a feed additive in animal diets for improved growth performance and overall health.

Alginate encapsulation has been widely used to protect and deliver bioactive compounds in various applications, including pharmaceuticals, nutraceuticals, and functional foods. The alginate matrix provides a protective barrier against environmental factors, such as heat, light, and pH, which can degrade the bioactive compounds. Additionally, the alginate matrix can control the release of the encapsulated compounds, allowing for sustained release and targeted delivery. In the case of the alginate-CaCl2 synbiotic nanocapsules, the high thermal stability observed suggests that the encapsulated synbiotic compounds are protected against high temperatures encountered during the pelleting process of animal feed production. This can be important for maintaining the viability and biological activity of the encapsulated probiotics and prebiotics [27]. Overall, alginate encapsulation can be a useful strategy to protect and deliver bioactive compounds in various applications. The addition of CaCl_2_ can enhance the stability of the alginate matrix, and the resulting complex can be used to encapsulate various compounds, including synbiotic formulations for animal feed applications.

The encapsulation of probiotics using alginate-CaCl_2_ nanocapsules maintained high viability of probiotics compared to non-encapsulated synbiotics after exposure of the synbiotics either to acidic conditions mimicking gastric conditions or storage for six months at room temperature. These results come in agreement with other studies that used alginate-based nanocapsules for the encapsulation of probiotics, supporting the relevance of such formulas to protect probiotics and maintain their viability under harsh conditions [23]. These findings can be due to the ability of the alginate-CaCl_2_ nanocapsules to encapsulate most probiotic cells inside its egg-box-like structure, providing adequate protection to a high number of probiotic cells against harsh conditions (mainly acidity and temperature), which is confirmed in our study by encapsulation of 99% of probiotic cells. Moreover, in our study, the phenolic compounds of prebiotic pomegranate peel may contribute to the improved viability of probiotic cells. Interestingly, phenolic compounds can selectively inhibit the growth of pathogenic bacteria without affecting the viability of probiotics [28]. For example, grapes and berries effectively inhibited the growth of pathogenic bacteria, but they had stimulatory effects on the probiotics [28]. Pomegranate juice, which is rich in ferulic, vanillic, and gallic acids, inhibited the growth of *Staphylococcus aureus*, *Pseudomonas aeruginosa*, and *Salmonella typhimurium* [29,30]. 

In this study, both alginate-CaCl_2_ synbiotic and free synbiotic showed antioxidant, antimicrobial, and antifungal activity, with higher activity for alginate-CaCl_2_ synbiotic. The antioxidant activity of the synbiotics can be mainly due to the effect of the phenolic compounds (mainly cinnamic, salicylic, chlorogenic, syringenic, caffeic, and gallic acids) of the prebiotic originated from pomegranate peel. Phenolic compounds—in particular, flavonoids and phenolic acids—are able to directly scavenge free radicals such as superoxide anion radicals (O^2−^) and hydroxyl radicals (OH^−^) [12]. In fact, the antioxidant activity of the synbiotics fabricated in this study is not only due to the action of phenolic compounds of prebiotic pomegranate but also may be due to the action of probiotics. Instantly, bacteria belonging to genera *Lactobacillus* and *Bifidobacterium* have been shown to decrease the levels of DPPH and 2,20-azino-bis (3-ethylbenzothiazoline-6-sulfonic acid) (ABTS) free radicals. Kim et al. [31] reported that the scavenging capacity of *Lactobacillus* is due to the action of bacterial exopolysaccharides, antioxidant enzymes, bioactive peptides, and manganese ions. These bacterial elements can directly scavenge free radicals, chelation of metal ions, and reduce the ascorbate autoxidation [32]. It is worth noting that the alginate-CaCl_2_ synbiotic showed antioxidant activity comparable to ascorbic acid and higher antioxidant activity than the free one. This can be due to two main reasons: the antioxidant activity of nanocapsule materials as alginate possesses antioxidant activity [33] and the protective role of nanocapsules for the synbiotic composites providing their longer and stronger biological activity.

One important aim of the present study is to find a natural and safe antimicrobial agent that can be used as an alternative to synthetic antibiotics. This is in line with the increasing global warning against the crisis of antimicrobial resistance and the related rapid and wide spreading of zoonotic diseases seriously threatening human and animal health. Moreover, inhibiting the growth of some mycotoxins-producing fungi becomes a crucial need for producing free mycotoxins foods and/or feeds [34]. The antimicrobial activity of synbiotics (nanoencapsulated or not) observed in this study against Gram-negative (*Escherichia coli* ATCC 8739, *Pseudomonas aeruginosa* ATCC 90274, and *Salmonella typhi* ATCC 6539) and Gram-positive bacteria (*Staphylococcus aureus* ATCC 6538), and fungus (*Aspergillus niger* and *Aspergillus flavus*) supports their relevance to these purposes. The antimicrobial activity of probiotics *lactobacilli* used in this study may be due to the production of secondary metabolites, such as bacteriocin, lactic acid, and hydrogen peroxide (intracellular ROS-mediated cell damages) [35]. For example, *Lactococcus lactis* produces bacteriocin nisin, which has substantial antimicrobial activity against bacterial pathogens and fungi, such as *Escherichia coli*, *Staphylococcus aureus*, *Pseudomonas* sp., *Candida albicans*, and *Aspergillus niger* [6]. Similarly, the probiotics *Lactobacillus plantarum*, *Lactobacillus paracasei*, and *Saccharomyces cerevisiae* have antimicrobial activity against a wide range of pathogens, as observed in this study and several previous studies [35]. Moreover, the antimicrobial activity of prebiotic phenolic compounds and their selectivity against the growth of pathogens may play a pivotal role in the pronounced antimicrobial activity of the synbiotics [28]. Finally, results of antimicrobial activity revealed the importance of the use of nanoencapsulation technology to fabricate more effective synbiotics, as alginate-CaCl_2_ synbiotics showed higher antimicrobial activity than free synbiotics. In fact, this superior effect may be due to many reasons: 1—improved viability of probiotics and protection of the prebiotic active components from rapid degradation; 2—sustained release of the active components of synbiotic; 3—the ability of synbiotic composites to diffuse through the gastrointestinal milieu due to their high surface area and small size. 

## 5. Conclusions

The present study aimed to develop a natural and safe alternative to synthetic antibiotics by combining pomegranate peel extract as a prebiotic source and a mixture of probiotic species in a synbiotic formula, resulting in an effective antioxidant and antimicrobial product. The use of nanoencapsulation technology, specifically alginate-CaCl_2_ nanocapsules with ionic gelation procedure, provided the synbiotic with superior physicochemical, biological, and industrial properties compared to the non-encapsulated form. These results highlight the potential of using these novel synbiotics as natural feed additives for livestock farming, which can help maintain animal health and productivity in a sustainable manner. Future in vivo studies using different farm animal models, such as ruminants and monogastrics, are needed to explore the effects of these tailored synbiotics on animal health and productivity.

## Figures and Tables

**Table 1:** Phenolic compounds of pomegranate peel ethanolic extract detected by high-performance liquid chromatography (HPLC).

Phenolic Compound	Concentration, µg/100 mL
Cinnamic acid	13.26
Salicylic acid	5.36
Chlorogenic acid	5.33
Syringenic	4.68
Catechol	3.1
Caffeic	2.45
Gallic	2.19

**Table 2:** Antioxidant activity of the nanoencapsulated and free synbiotics assessed by the 2,2-diphenyl-1-picrylhydrazyl (DPPH) scavenging ability compared to ascorbic acid.

Concentration, µg/mL	DPPH Scavenging Activity, %		
	Nanoencapsulated Synbiotic	Free Synbiotic	Ascorbic Acid
1.95	41.2 ^a^	1.77 ^b^	41.7 ^a^
3.90	47.7 ^a^	3.5 ^b^	45.8 ^a^
7.81	56.5 ^a^	7.1 ^b^	56.3 ^a^
15.62	63.8 ^a^	14.2 ^b^	64.2 ^a^
31.25	70.6 ^a^	24.8 ^b^	71.2 ^a^
62.5	77.6 ^a^	47.8 ^b^	78.0 ^a^
125	85.3 ^a^	56.2 ^b^	86.4 ^a^
250	90.3 a	62.2 ^b^	92.7 ^a^
500	94.3 ^a^	68.7 ^b^	94.5 ^a^
1000	96.2 ^a^	74.1 ^b^	97.0 ^a^
SEM	9.56	9.78	9.73
Half-maximal inhibitory concentration			
IC 50	3.96 ± 0.42 ^b^	65.75 ± 2.14 ^a^	4.08 ± 0.79 ^b^

^a,b^ Means in the same row followed by uncommon superscript letters are significantly different (*p* < 0.05).

**Table 3:** Antibacterial and fungal activity of nanoencapsulated synbiotics and free synbiotics against some Gram-positive and Gram-negative microbial strains compared to gentamicin (antibacterial drug) and fluconazole (antifungal drug) antibiotics.

Treatment	Inhibition Zone, mm					
	Bacterial Strains				Fungal Strains	
	*Escherichia* *coli* (ATCC 8739)	*Staphylococcus aureus* (ATCC 6538)	*Pseudomonas aeruginosa* (ATCC 90274)	*Salmonella typhi* (ATCC 6539)	*Aspergillus* *niger*	*Aspergillus* *flavus*
Nanoencapsulted synbiotic	19.00 ^a^	20.67 ^a^	20.33 ^a^	19.33 ^a^	16.67 ^b^	17.00 ^b^
Free synbiotic	13.00 ^c^	16.00 ^b^	15.33 ^b^	19.00 ^a^	14.67 ^c^	13.33 ^c^
Gentamicin	17.00 ^b^	18.00 ^a,b^	19.33 ^a^	15.67 ^b^	-	-
Fluconazol	-	-	-	-	26.00 ^a^	21.00 ^a^
SEM	0.374	0.470	0.795	1.13	1.03	1.73
*p*-value	<0.001	<0.001	<0.001	<0.001	<0.001	<0.001

^a,b,c^ Means in the same column followed by uncommon superscript letters are significantly different (*p* < 0.05).

## Data Availability

Data of this study is confidential.
